# Office-based Air-Fluid Exchange for Diabetic Post-Operative Vitreous Cavity Hemorrhage

**Published:** 2019

**Authors:** Alice W. BEHRENS, Sami H. UWAYDAT, Joshua S. HARDIN, Ahmed B. SALLAM

**Affiliations:** 1 Jones Eye Institute, Department of Ophthalmology, University of Arkansas for Medical Sciences, Little Rock, Arkansas, USA

**Keywords:** Vitreous Hemorrhage; Air-Fluid Exchange; Diabetic Retinopathy

## Abstract

Post-operative vitreous cavity hemorrhage (POVCH) is observed in 6-75% of eyes undergoing pars plana vitrectomy (PPV) for proliferative diabetic retinopathy (PDR). We describe our technique for office-based Air fluid exchange (AFX) in the treatment of POVCH. Sixteen eyes (15 patients) with PDR and POVCH undergoing office-based AFX between January 2006 and November 2016 were retrospectively identified. The pre- and post- procedure visual acuity (VA) and complications were compared between eyes with and without traction retinal detachment (TRD). Medicare charges for office-based AFX versus PPV were also analyzed. Mean (± standard deviation [SD]) age at the time of AFX was 55.31 (± 8.02) years. Nine eyes (56.25%) had TRD prior to PPV and 11 eyes (68.75%) were pseudophakic. The improvements in mean (±SD) logMAR VA at the last postoperative visit (3 - 8 months) were 1.38 (± 0.99), 0.82 (± 0.91) and 2.09 (± 0.53) in all eyes, TRD eyes and non-TRD eyes, respectively. Complications included cataract progression, hypotony, and recurrence of TRD and ghost cell glaucoma. The total cost of outpatient AFX was $1,409.59 less than that of PPV. Office-based AFX is a cost-effective alternative treatment for non-clearing diabetic POVCH with an acceptable risk profile.

## INTRODUCTION

Postoperative vitreous cavity hemorrhage (POVCH) is a relatively common complication, observed in up to 75% of eyes following pars plana vitrectomy (PPV) in the setting of proliferative diabetic retinopathy (PDR) [1-6]. While most cases of POVCH result in spontaneous clearing, repeat PPV is required in 10-38% of cases where POVCH persists [1, 2, 4].

Air fluid exchange (AFX) is an alternative treatment approach to PPV for POVCH that can be undertaken in an office-based setting [7-10]. There is a paucity of contemporary literature on the efficacy and safety of office-based AFX for POVCH, with earlier studies describing cataract formation, iris neovascularization and anterior hyaloid fibrovascular proliferation as main complications [8-10]. As patient characteristics have changed with modern vitrectomy instrumentation, earlier cataract surgery, more effective laser treatment and integration of intravitreal anti-vascular endothelial growth factor (VEGF) injections into the treatment of diabetic macular edema and PDR, there is a need for contemporary re-evaluation of this treatment approach. With a renewed emphasis on the cost of healthcare, a modern-day comparison of the cost effectiveness of office-based AFX versus PPV is valuable. 

We conducted a retrospective chart review study of eyes that underwent office-based AFX for POVCH at our institution. We described their visual outcomes and post-procedure complications and presented a cost-comparison of office-based AFX versus PPV.

## METHODS

Data Categorization

We retrospectively reviewed electronic charts of adult patients (age >18 years) who underwent office-based AFX for the treatment of POVCH between January 2006 and November 2016 at the University of Arkansas for Medical Sciences, State of Arkansas, United States. Patients were identified through current procedure terminology (CPT) code search in the electronic medical record (Epic, Epic System Corporation, Verona, WI). We collected data including age, sex, laterality, visual acuity (VA), duration of post-AFX follow-up, time interval from initial PPV to POVCH, duration of POVCH, post-procedure complications, history of tractional retinal detachment (TRD) and crystalline lens status. Visual acuity, recorded as counting fingers (CF), hand motions (HM), light perception (LP) and no light perception (NLP) were converted to 2.1, 2.4, 2.7, and 3.00 logMAR, respectively [11]. We included both eyes when both had undergone the procedure and excluded eyes with less than 3 months of follow-up and those with vitreous cavity hemorrhage secondary to causes other than PDR. Visual improvement after AFX was defined as a gain of 0.3 LogMAR or more (approximately 2 Snellen lines) [12].

This study was conducted in compliance with the Declaration of Helsinki and ethical approval was obtained from ethical committee for research of University of Arkansas for Medical Sciences. 

Office-based Air-Fluid Exchange Technique

Our technique for office-based AFX differs slightly from those previously described [7-10]. First, B-scan ultrasonography is performed to exclude a retinal detachment. Then, the patient is placed in a reclined position. After topical anesthesia, the eye is prepped with a 5% povidone iodine solution and a lid speculum is placed. Using sterile technique and an air filter, a 10 mL syringe is filled with sterile air. A half-inch 30-gauge needle is then attached and inserted superotemporally (nondependent position) through the pars plana into the vitreous cavity. A second 30-gauge needle, attached to a 10 mL syringe, is then inserted through the pars plana at 6 O’clock (dependent position) into the vitreous cavity. Injection of air through the nondependent syringe is performed, matching the rate of aspiration of the hemorrhage through the dependent syringe. The procedure is continued until air is observed in the dependent syringe and is typically performed in less than 10 minutes.

Main Outcome Measures

Visual acuity and post-procedure complications were considered as primary outcomes for this study. For analyses, visual outcome data was further segregated by history of TRD and the time to onset and observation duration of POVCH. Costs were analyzed using Medicare fee data and CPT codes which were used to calculate facility and non-facility fees, using a standard 2016 relative value unit (RVU) cost of $35.8887 per RVU [12, 13].

Anesthesia fees were calculated on base and time units with a conversion factor of $22.0454 [14].

Continuous variables were summarized as mean and range values and additional statistical analyses were performed using statistics calculators of the online forum Social Science Statistics. The Wilcoxon signed-rank test was used for the assessment of VA, the Mann-Whitney U test for continuous data, the Fisher exact test for categorical data and the Spearman’s Rho test for correlations. A p-value less than 0.05 was considered as statistically significant difference. 

## RESULTS

Sixteen eyes of 15 patients were included in this study. Demographic and visual outcome data are shown in Table 1. Of the 16 eyes in the study, 9 eyes (56.25%) had persistent (onset < 3 months after PPV) and 7 eyes (43.75%) had recurrent (onset > 3 months after PPV) POVCH. Eleven eyes (68.75%) were pseudophakic and 5 (31.25%) were phakic. Four eyes (25%) had received intravitreal anti-VEGF or steroid injections prior to AFX and 5 (31.25%) received such treatments post-AFX. All eyes underwent at least one session of panretinal photocoagulation (PRP) before or soon after AFX. The mean (± standard deviation [SD]) duration of post-AFX follow-up was 6.19 (± 1.34) months (median: 6.5 months, range: 3.5 - 8 months) and three eyes (18.75%) required a second AFX procedure. 

Prior to AFX, the mean VA (± SD) was 2.38 (± 0.17) logMAR (~ HM) (range: 2.1 to 2.7 logMAR). After AFX, the mean VA (±SD) was 1.31 (± 0.97) LogMAR (~ 20/400 Snellen VA) at 3-month post procedure visit and 1.04 (± 0.96) LogMAR (~ 20/200 Snellen VA) at the last post-procedure visit which was recorded between 3 and 8 months after AFX. Of 16 eyes in this study, 14 showed improvement in VA after AFX. The mean improvement in VA at 3 months was 0.93 logMAR units (~ 6 Snellen lines), which was statistically significant (P = 0.004, Wilcoxon signed-rank test). The mean improvement in VA at the final follow-up was 1.38 logMAR units (~ 9 Snellen lines) and this was also statistically significant (p = 0.001, Wilcoxon signed-rank test).

Prior to PPV surgery, 8 eyes (50%) had TRD with macular involvement and one eye had TRD without macular involvement. The mean (±SD) improvement in VA was 0.82 (± 0.91) logMAR units with TRD and 2.09 (± 0.53) logMAR units without TRD (p = 0.015, Mann-Whitney test). Four of the 9 eyes (44.4%) with TRD and all 7 eyes without TRD achieved a VA of 1.3 logMAR (~ 20/400 Snellen VA) or better (P = 0.034, Fisher’s exact test). The time to onset (following PPV) and observation duration of POVCH were negatively associated with post-AFX improvement in VA significantly (P = 0.02 and P = 0.02, respectively, Spearman Rho’s test). No intra-procedural complications were encountered. Post-procedure complications are described in Table 2 including worsening of cataract in all 5 phakic eyes, ocular hypertension (intraocular pressure [IOP] > 22mmHg) in 3/16 eyes (18.75%), hypotony (IOP < 6mmHg) in 1/16 eyes (6.25%), recurrence of TRD in 2/9 eyes (22.22%), iris neovascularization in 1/16 eyes (6.25%) and ghost cell glaucoma in 1/16 eyes (6.25%). Four of the five phakic eyes (80%) required cataract surgery with a mean time to cataract surgery of 3 months (median: 3 months, range: 1.5 - 6 months) following AFX. Three eyes (18.75%) required repeat PPV after AFX for recurrence of TRD and new retinal breaks. Two of these three eyes showed no improvement in VA after repeat PPV. The costs associated with office-based AFX and repeat PPV are described in Table 3. The charges for office-based AFX, performed in a hospital-associated outpatient setting, include non-facility fee of $737.15 [13, 14].

For AFX performed in a non-hospital associated outpatient clinic, no facility fee is applied and the overall charge for AFX consists only of a professional fee of $737.15. The fees associated with a 30-minute PPV in the operating room in an ambulatory surgery center (ASC) not owned by operating surgeons include anesthesia fee of $176.36 [15], and hospital/ambulatory surgery center facility fee of $1750.01 [14] and surgeon professional fee of $916.60, resulting in a total of $1092.96.

**Table 1:** Patient Characteristics Before and After Air Fluid Exchange Procedure

Case	Age, Race and Gender		Persistent vs. Recurrent POVCH	Post-PPV Time Interval (month)	POVCH Duration (week)	Pre-AFX logMAR VA	No. AFX Procedures	Repeat PPV/ indication	Lens Status	TRD	PRP	Intravitreal Anti-VEGF / Steroid (pre/post- AFX)	Post-AFX logMAR VA (most recent)	Complications	Follow- Up Duration (month)
1	67, AAF		Recurrent	23	1.28	2.4	1	No	Pseudophakic	No	Yes	Bevacizumab x 2 (post)	0.5	None	6
2	65, CF		Persistent	2	4.28	2.7	1	No	Phakic	No	Yes	Bevacizumab x 1 (post)	0	Cataract	7.5
3	59, CM		Persistent	1.16	5	2.4	1	Yes (new retinal breaks, ERM)	Phakic	Yes	Yes	Bevacizumab x 1 (pre) Bevacizumab x 1 (post)	2.1	NVI, Cataract	6.5
4	64, AAF		Recurrent	6	9	2.7	1	No	Pseudophakic	Yes	Yes	None	3.0	None	7.5
5	41, AAF		Recurrent	6	4.28	2.4	2	No	Pseudophakic	No	Yes	None	0.4	None	7
6	53, AAM		Persistent	2	4.28	2.1	1	Yes (recurrent VH, ERM)	Pseudophakic	Yes	Yes	None	2.1	Recurrent TRD	6.5
7	47, AAM		Persistent	9	39.4	2.4	1	Yes (new TRD, ERM)	Phakic	Yes	Yes	None	2.1	Recurrent TRD, hyphema, cataract	7.5
8	48, AAF		Recurrent	10	2	2.4	1	No	Phakic	Yes	Yes	None	1.3	Recurrent TRD, cataract	6
9	53, CF		Persistent	0.23	1	2.4	1	No	Pseudophakic	Yes	Yes	None	0.2	None	6.5
10*	52, AAM		Persistent	0.23	1	2.7	2	No	Pseudophakic	No	Yes	Bevacizumab x 1 (pre)	0.6	None	4.5
11*	52, AAM		Persistent	0.13	2	2.4	1	No	Pseudophakic	Yes	Yes	Bevacizumab x 1 (pre)	0.4	Ghost cell glaucoma	7
12	54, HF		Recurrent	15	4.28	2.4	2	No	Pseudophakic	Yes	Yes	None	1.3	None	3.5
13	46, CF		Recurrent	17	3	2.4	1	No	Pseudophakic	Yes	Yes	None	2.1	None	3.5
14	57, AAF		Persistent	0.93	3	2.1	1	No	Pseudophakic	Yes	Yes	Aflibercept x 3 (pre), Aflibercept x 1 (post)	0.6	None	5
15	58, AAM		Persistent	0.07	1.71	2.7	1	No	Phakic	No	Yes	Bevacizumab x 1 (post)	0.1	Hyphema, cataract	6.5
16	69, CM		Persistent	0.07	3	2.4	1	No	Pseudophakic	No	Yes	None	0.2	none	8

Abbreviations: AAF: African American female; AAM: African American male; AFX: air-fluid exchange; Anti-VEGF: Anti–vascular endothelial growth factor; CF: Caucasian female; CM: Caucasian male; ERM: epiretinal membrane; HF: Hispanic female; NVI: neovascularization of iris; POVCH: post-operative vitreous cavity hemorrhage; PPV: pars plana vitrectomy; PRP: panretinal photocoagulation; TRD: tractional retinal detachment; VA: visual acuity; logMAR: Logarithm of the minimum angle of resolution; V.S: Versus; VH: vitreous hemorrhage. *Study eyes number 10 and 11 are from the same patient.

**Table 2:** Post air-fluid exchange (AFX) Complications.

Complications	No. of eyes
** Hypotony**	1 (6.7%)
Elevated IOP (1wk post AFX)	
** IOP** ** (30-40) mmHg**	1 (6.7%)
** IOP** ** (22-30) mmHg**	2 (13.3%)*
** Hyphema**	2 (13.3%)
** Worsening** ** cataract requiring surgery**	5/5 (100%)
** Cataract** ** surgery post AFX**	5 (100%)
** Recurrent** ** TRD**	3/9 (33.3%)
** NVI**	1/16 (6.7%)
** Ghost** ** cell glaucoma**	1/16 (6.7%)

Post air-fluid exchange complications include hypotony, elevated intraocular pressure (IOP), hyphema, cataract progression, recurrent traction retinal detachment (TRD) and neovascularization of iris (NVI). Numbers of eyes with each complication are listed. * Intraocular pressure (IOP) in all three cases after air-fluid exchange was lower than pre-air-fluid exchange IOP. Abbreviation: AFX: air-fluid exchange; wk: week; mmHg: millimetre of mercury.

**Table 3:** Cost Analysis of Air-Fluid Exchange performed in Clinic Versus Pars Plana Vitrectomy Performed in an Operating Room at an Ambulatory Surgery Center.

	AFX (CPT code 67025)	PPV (CPT code 67036 without modifier)
Total RVU	20.54	25.54
Professional fee		
** price**	$737.15	$916.60
** limiting charge**	$805.34	$1001.38
ASC payment	N/A	$1750.01
Anesthesia fee (30-minute)	N/A	$176.36
Total fees	$737.15	$2843.97
Total fees limits	$ 805.34	$ 2927.75

Total revenue value unit (RVU) is the sum of work RVU, facility or non-facility RVU and malpractice RVU, based on 2017 Medicare physician fee schedule. Professional fee is the amount of reimbursement for the physician based on total RVU multiplied by national conversion factor 35.8887, and the limiting charge is the upper limit payment for physicians, which is usually 115% of the professional fee, as provided by the 2017 Medicare physician fee schedule using the fee of national payment schedule as the Medicare administrative contractor option. Ambulatory surgery center (ASC) payment Anesthesia fee is calculated based on base unit of CPT code 00145 for 30 minutes of vitreoretinal surgery. Total fees are the sum of hospital or ambulatory surgery center professional fee, ASC facility fee and anesthesia fee. Total fees limits are the sum of ASC payment and anesthesia fee. Abbreviations: AFX: air-fluid exchange; PPV: Pars Plana Vitrectomy; CPT: current procedural terminology.

## DISCUSSION

We described visual outcomes, post-procedure complications and relative cost of office-based AFX for POVCH in the setting of PDR. We found a notable improvement in mean VA of 1.38 logMAR units (~ 9 Snellen lines), with 14 of 16 eyes showing improvement. Eyes with TRD showed less improvement than those without it, with a gain of 0.82 versus 2.09 logMAR units, respectively. We found negative associations between vision improvement and the time to onset and the observation duration of POVCH. We calculated a difference in expenses up to $2121.45 when comparing office-based AFX to a 30-minute PPV (CPT 67036) in the operating room.

The visual improvement we found with office-based AFX was not substantially different from those previously described. A study by Martin and McCuen reported a median pre-AFX VA of 2.45 logMAR (HM) and a median post-AFX visual acuity of 0.30 logMAR (20/40 Snellen VA) for AFX alone [10]. Another study by Han, et al. found median pre- and post-AFX visual acuities of 2.4 logMAR (HM) and 1.20 logMAR (20/300 Snellen VA), respectively [9]. Our median pre- and post-AFX visual acuities were 2.4 logMAR (HM) and 0.95 logMAR (20/200 Snellen VA), respectively. In a study by Blankenship examining post-AFX visual outcomes, 12/19 eyes (63%) improved to 0.95 logMAR (20/200 Snellen VA) or better with AFX [8]. In our study, 8/16 eyes (50%) improved to this level of vision. We found better outcomes in eyes without TRD and those with a shorter time to onset and observation duration of POVCH, likely secondary to better pre-AFX vision potential in these eyes.

Regarding the efficacy of AFX as a treatment for POVCH, it is also important to assess the number of eyes requiring repeat AFX procedures and / or PPV for a non-clearing hemorrhage. In the study by Martin and McCuen, the mean number of AFX procedures performed per patient was 1.5, with 40% requiring a repeat vitrectomy [10]. In the study by Han et al., the mean number of AFX procedures per eye was 1.75, with 25% eyes requiring a repeat vitrectomy [9]. And in our study, the mean number of AFX procedures per eye was 1.2, with 3 of 16 eyes (19%) requiring a repeat vitrectomy. 

We observed a lower incidence of post procedure complications with AFX than previously described [7-10]. Prior studies have noted cataract to be a major complication of AFX, with cataract development reported in up to 59% of eyes [8-10]. However, 84-92% of the eyes in these studies were phakic at the time of AFX versus only 31% in our study. Other complications noted previously include iris neovascularization (12-26.3%), anterior hyaloidal fibrovascular proliferation (5-12.5%), neovascular glaucoma (12-37%) and ghost cell glaucoma (5%) [8-10]. notably the risk of fibrovascular complications in our study was substantially lower; iris neovascularization in 1 eye (6.25%), ghost cell glaucoma in 1 eye (6.25%) and no cases of anterior hyaloid fibrovascular proliferation. Over the past 20 years, cataract surgery has become more prevalent in the United States [16, 17]. In addition to negating a concern for cataract development in these patients after PPV or AFX, pseudophakia offers a benefit over aphakia for decreased VEGF circulation from the posterior segment and thus possibly a reduced risk of neovascular complications [18]. Modern vitrectomy may have contributed to decreased risk of iris neovascularization, neovascular glaucoma and anterior hyaloid fibrovascular proliferation found in our study. Compared to the early era of vitrectomy, small gauge, high speed vitrectomy instruments and improved endolaser systems enable more thorough relief of vitreoretinal traction and treatment of retinal ischemia [19-21]. Further, the reduced risk of fibrovascular complications may also be related to the current trend of improved preoperative control of PDR with laser and intravitreal anti-VEGF treatments.

With recent concern for the rising cost of healthcare with an aging population, physicians are more mindful of care-associated costs. Office-based AFX is a clinic-based procedure, which takes less time and planning than does a transfer to the operating room. In a carefully selected patient population recurrent vitreous cavity hemorrhage after PPV in the absence of TRD and pseudophakic eyes, office-based AFX is an effective and cost effective first line procedure to clear the hemorrhage. The success rate of clearing vitreous cavity hemorrhage at 3-8 months post-procedure is 81% (13 of 16 eyes) with single AFX (Table 1). Having the AFX performed in the ophthalmologist clinic, ASC or hospital facility fee as well as anesthesia fee are non-existing. Based on the 2017 Medicare physician fee schedule, ASC payment rate and anesthesia fee schedule [13, 14], the procedure payment for office-based AFX (CPT 67025) was 2104.93 less than a 30-minute PPV performed in ASC (CPT 67036) (Table 3).

This study is limited by its small size, retrospective design and short follow-up time. It could also be subject to selection bias, as cases of worse POVCH or those with more significant preoperative TRD may have been treated with repeat PPV. This may explain the small number of cases in our study. Also, the differences in the reporting of visual outcomes make comparison to previous studies difficult. Follow-up time past 3 months was inconsistent and we reported final VA measured at 3 to 8 months as our primary visual outcome. Office-based AFX does not allow physicians to address worsening traction as a cause for bleeding and will not allow for the removal of large clots. Finally, outcomes may have also been affected by pre-procedure vision potential. Despite these limitations, our study is a useful examination of office-based AFX in the setting of POVCH and the use of contemporary vitrectomy instruments and anti-VEGF treatments for diabetic retinopathy. It also offers a modern-day cost comparison to repeat PPV.

## CONCLUSIONS

In conclusion, office-based AFX is a useful and cost-conscious alternative treatment for patients with POVCH secondary to PDR, particularly in the absence of TRD and pseudophakic patients. Office based AFX is also useful for patients who have multiple recurrent POVCH despite multiple PPV and endolasers, as well as for those who are high risk patients to undergo general anesthesia or retrobulbar block. We found that improvement in mean VA of 1.38 logMAR units (~ 9 Snellen lines) was comparable to previous studies, with greater improvements noted in patients without TRD and those with earlier onset and shorter duration of observation.
